# Decoupling photo- and thermoperiod by projected climate change perturbs bud development, dormancy establishment and vernalization in the model tree *Populus*

**DOI:** 10.1186/s12870-018-1432-0

**Published:** 2018-10-05

**Authors:** Päivi L. H. Rinne, Laju K. Paul, Christiaan van der Schoot

**Affiliations:** Faculty of Biosciences, Department of Plant Sciences, Norwegian University of Life Sciences, Christian Magnus Falsens vei 18, 1432 Aas, Norway

**Keywords:** Callose, Chilling, Climate change, Dormancy Sphincter Complexes, Ecotype, Flowering, Gibberellin, 1,3-β-Glucanases, Plasmodesmata, Terminal bud

## Abstract

**Background:**

The performance and survival of deciduous trees depends on their innate ability to anticipate seasonal change. A key event is the timely production of short photoperiod-induced terminal and axillary buds that are dormant and freezing-tolerant. Some observations suggest that low temperature contributes to terminal bud initiation and dormancy. This is puzzling because low temperatures in the chilling range universally release dormancy. It also raises the broader question if the projected climate instabilities, as well as the northward migration of trees, will affect winter preparations and survival of trees.

**Results:**

To gauge the response capacity of trees, we exposed juvenile hybrid aspens to a 10-h short photoperiod in combination with different day/night temperature regimes: high (24/24 °C), moderate (18/18 °C), moderate-low (18/12 °C) and low (12/12 °C), and analysed bud development, dormancy establishment, and marker gene expression. We found that low temperature during the bud formation period (pre-dormancy) upregulated dormancy-release genes of the gibberellin (GA) pathway, including the key GA biosynthesis genes *GA20oxidase* and *GA3oxidase*, the GA-receptor gene *GID1*, as well as GA-inducible enzymes of the 1,3-β-glucanase family that degrade callose at plasmodesmal Dormancy Sphincter Complexes. Simultaneously, this pre-dormancy low temperature perturbed the expression of flowering pathway genes, including *CO*, *FT*, *CENL1, AGL14*, *LFY* and *AP1*. In brief, pre-dormancy low temperature compromised bud development, dormancy establishment, and potentially vernalization. On the other hand, a high pre-dormancy temperature prevented dormancy establishment and resulted in flushing.

**Conclusions:**

The results show that pre-dormancy low temperature represents a form of chilling that antagonizes dormancy establishment. Combined with available field data, this indicates that natural *Populus* ecotypes have evolved to avoid the adverse effects of high and low temperatures by initiating and completing dormant buds within an approximate temperature-window of 24-12 °C. Global warming and erratic temperature patterns outside this range can therefore endanger the successful propagation of deciduous perennials.

**Electronic supplementary material:**

The online version of this article (10.1186/s12870-018-1432-0) contains supplementary material, which is available to authorized users.

## Background

Deciduous trees in temperate areas have a surprisingly short growing season. At northern latitudes, most of their lives are spent in a leafless state and exposed to low temperatures. Preparation for the winter requires multiple developmental adjustments, which are informed by the sequential decline in photoperiod and temperature. Photoperiod changes predictably during the Earth’s motion around the sun, and photoperiodic ecotypes exploit this regularity as an unambiguous measure of seasonal progression. The gradual shift in photoperiod and solar angle, and the corresponding change in radiation, drive the annual thermal cycle that determines the latitudinal and altitudinal distribution of plants [1]. While photoperiod regulates the initiation and development of endo-dormant buds, hereafter referred to as dormant buds, subsequent exposure to low non-freezing temperatures releases dormancy and enhances freezing-tolerance [2]. Currently, increased radiative forcing is raising the average global temperature, although the high northern latitude is projected to warm disproportionally [3], a phenomenon referred to as Arctic Amplification [4]. As the shifting climate is outpacing tree migration [5–7], this will significantly affect both temperate and boreal forests, which harbour half of the world’s trees [8–10].

Many tree species are adapted to respond to critical short photoperiods, which are longer toward the North, resulting in shorter growing seasons at high latitudes [11]. For example, *Betula* and *Salix* populations that are adapted to latitudes in the far North, initiate terminal buds (TBs) at a surprisingly long 22-h photoperiod, whereas southern ecotypes continue to grow until daylengths decrease to 15 h [12]. For this reason, ecotypes do not prosper far outside their latitude. Changes in the annual thermal cycle are expected to challenge the adaptive mechanisms on which these ecotypes depend. As global warming tends to drive the geographic distribution of species towards the North, they could initiate winter preparations belatedly, according to the critical photoperiod of their origin, falling victim to arriving frost.

On the other hand, at their current location, trees will not be able to profit from a longer growing season, due to their photoperiodic responsiveness, and might be outcompeted by invasive species. Moreover, it is unknown how the elevated temperatures will affect the seasonal cycle of perennials in general. Inherent to the global rise in temperature are climate instabilities [3, 4]. For example, in northern birch dormancy is released relatively early in autumn, well before winter arrives [13]. Subsequent long warm spells in late autumn could trigger bud flushing and de-acclimation, rendering meristems vulnerable to frost [14, 15].

Controlled climate room experiments have demonstrated that photoperiod alone is sufficient to induce dormant buds at growth-promoting temperatures, and that subsequent chilling is required to release dormancy and enhance freezing tolerance [2, 13, 16, 17]. The role of temperature in dormancy establishment has remained controversial. Classic literature suggests that some trees, such as *Populus, Malus* and *Acer*, lose the capacity to form dormant TBs under low temperature conditions [18–21]. This would make successful north ward migration unlikely. Contrary to this, other studies propose that low temperature on its own could induce the establishment of dormant buds or contribute to it [22–27].

One reason for this ambiguity about the role of low temperature could be that bud formation is confused with dormancy. For example, in proleptic hybrid aspen, para-dormant and dormant axillary buds (AXBs) are structurally similar, but shoot decapitation can only activate para-dormant AXBs [28, 29]. TBs can also form temporarily under stress without establishing dormancy [12, 19, 30]. Even tropical trees show this behaviour during episodic growth, in response to drought, or during the alternation of elongation growth and branching [19, 31, 32]. Thus, conclusive assessment of whether buds are dormant, i.e. intrinsically arrested, requires tests in which buds are freed from apical dominance and correlative inhibition. Presently, tests should also include analyses of key marker genes of dormancy [28, 29, 33, 34]. The claim that low temperature in the chilling range can induce dormancy or contribute to its establishment is puzzling, because it is a universal cause of dormancy release [35–37], and there is no explanation of how low temperature could both induce and antagonize the same state. The manner in which temperature intersects with the photoperiodic pathway during bud initiation and dormancy therefore requires further investigation.

The photoperiodic response that results in TB formation is well documented. For example, *Betula* and *Populus* monitor the declining photoperiod by means of light-sensitive phytochromes in the leaves [25, 38]. These pigments provide input to a circadian clock that transcriptionally regulates expression of the floral promoter *CONSTANS* (*CO*) and the key integrator gene *FLOWERING LOCUS T* (*FT*) [25]. *Populus* possesses two functionally distinct paralogs, *FT2* and *FT1*, which promote vegetative growth and flowering, respectively [34, 39, 40]. Exposure to short photoperiod downregulates *FT2* in the leaves, and transiently upregulates the gene *CENTRORADIALIS-*like1 (*CENL1*) at the rib meristem to support TB formation [28, 34]. Thereby, the apex switches to the developmental program of AXBs, both structurally and molecularly. When TBs and the young AXBs complete their development and establish dormancy, *CENL1* expression ceases. In contrast, para-dormant AXBs that develop under long photoperiod maintain a high level of *CENL1* expression and are poised for vegetative growth, only requiring removal of apical dominance [28]. During chilling-induced dormancy release, *FT1* transcription is hyperinduced, but *CENL1* is not [40]. As these genes are central in the regulation of vernalization and floral transition [39, 41, 42], this suggests that low temperature not only releases dormancy, but may also promote vernalization of dormant buds. To clarify the role of temperature in dormancy establishment, it is important to assess how exposure to low temperature during the bud development phase (hereafter referred to as pre-dormancy) interferes with the transcriptional patterns of key dormancy-cycling and vernalization genes.

At the morphogenetic level, TB development follows a predictable scenario. In the active shoot apical meristem (SAM) all cells participate in a gated symplasmic network that functionally and dynamically integrates cellular activities. We proposed a model in which the SAM is the organizer of primary development as well as the specific locus of dormancy [43–45]. We showed that under short photoperiod, callosic Dormancy Sphincter Complexes (DSC) close off plasmodesmata. These specialized DSC act as circuit breakers in the symplasmic circuitry of the SAM, blocking signal exchange as well as metabolic and electrical coupling, and dissipating supracellular surveillance and coordination [43, 46–48]. Subsequently, cellular activities diminish as membrane potentials drop, and metabolism and gene expression change as part of the acclimation process. Remarkably, this symplasmic shutdown is reversed by chilling-induced removal of DSC callose. As a result, the symplasmic network is restored, enabling renewed cell-cell communication once rising temperature gears up metabolism [16, 40].

The mechanisms by which photoperiod and chilling trigger the symplasmic alterations in the SAM have been investigated in detail in birch and hybrid aspen [16, 34, 40, 49]. Since a main constituent of the DSC is callose [16], the balance between 1,3-β-glucan synthases and 1,3-β-glucanases [50] is crucial in establishing and releasing dormancy [40, 41, 51]. The callose-hydrolysing 1,3-β-glucanases (family GH17) are diversified into an α-, β- and γ-clade [40, 52]. In hybrid aspen, the studied growth-related α-clade enzymes are downregulated, whereas γ-clade members are upregulated during bud formation as well as during chilling-induced dormancy release [29, 40]. Crucially, the growth-related α-clade members are inducible by GA_4_, while bud-related γ-clade members are responsive to GA_3_. The γ-clade enzymes localize at lipid bodies that accumulate in developing buds, and are displaced by chilling to plasmodesmata where they can remove callose [40, 43]. As this cellular mechanism is recruited by chilling, this raises the question if low temperature in the pre-dormancy phase could do the same, thereby counteracting installation of DSC and antagonizing establishment of dormancy.

The role of low temperature in dormancy cycling is often discussed in terms of its signal value. However, both photosynthesis and phloem transport are sensitive to low temperatures [1, 53], which are likely to diminish the development of growing buds. Therefore, producing TBs before temperatures drop seems a superior strategy, as it allows diversion of photosynthates from growing points to buds and other reserve stores well before cold halts photosynthesis, induces leaf abscission, and renders sieve tubes in the shoot dysfunctional [54, 55].

The question if temperature influences the photoperiodic response that results in bud dormancy has a certain urgency in the context of climate change and the inherently unstable weather patterns. Decoupling of photo- and thermoperiod might present trees with conflicting environmental information, which could putatively diminish tree performance and survival in the long term. It is conceivable that unseasonal low and high temperatures could adversely affect the establishment of dormant buds. To address these possibilities, we exposed juvenile hybrid aspens (*Populus tremula* x *P. tremuloides*) to short photoperiod at four different day/night (D/N) temperature regimes: high (D/N 24/24 °C), moderate (D/N 18/18 °C), moderate-low (D/N 18/12 °C), and low (D/N 12/12 °C). We investigated TB initiation, AXB development, and bud dormancy establishment as well as the expression of marker genes. A main finding is that even mildly low temperatures (12 °C) during the bud-formation phase adversely affect the scheduling of the distinct phases of the dormancy cycle. Importantly, it diminishes bud-quality, prematurely triggers genes of the dormancy-release mechanism, and perturbs transcription of key vernalization pathway genes. Furthermore, at a high temperature (24 °C), the developing TBs flush regularly, and fail to establish dormancy. Our analyses of the critical photoperiod of given ecotypes in combination with local weather data support the hypothesis that natural *Populus* ecotypes have evolved to produce viable dormant TBs and AXBs within an approximate 24-12 °C temperature window, before temperatures reach chilling levels.

## Results

### Temperature modifies shoot elongation

Shoot elongation under long photoperiod involves a number of apical internodes that elongate simultaneously, albeit at different rates [34]. Subsequent exposure to short photoperiod halts the production of new internodes, and eventually shoot elongation. To investigate if and how temperature influences this process, we monitored the response of two categories of internodes: (1) internodes elongating already under long photoperiod, and (2) internodes that were still in their incipient phase, including those that arose during the treatment.

**Fig. 1:**
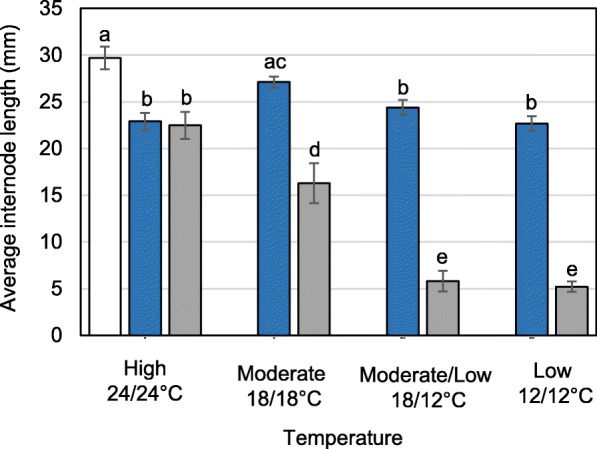
Internode elongation under short photoperiod and different temperature regimes, high (24/24 °C; D/N), moderate (18/18 °C; D/N), moderate/low (18/12 °C; D/N) and low (12/12 °C; D/N; day/night). White bar represent final internode length under long photoperiod at 18/18 °C. Blue bars represent the final length of internodes, excluding the last three, under short photoperiod and the indicated temperature regime. Grey bars represent the final internode length of the three internodes that were in the incipient stage during the start of short photoperiod. Different letters refer to statistically significant differences (*p* < 0.001) (values are means ± SE, *n* = 15 per data point)

Internodes of the first category (long photoperiod, 18 h; 18/18 °C) slightly reduced elongation under subsequent short photoperiod relative to those that remained under long photoperiod (Fig. 1). Although the reduction was not statistically significant, it could reflect the shortening of the period plants could photosynthesize. A statistically significant reduction of approx. 17% (Fig. 1) was measured after lowering the night-temperature (18/12 °C), reflecting reduced night-time phloem transport from source leaves to internode sinks. Lowering also the day-temperature reduced elongation further to 24% (Fig. 1), possibly by diminishing photosynthesis. In brief, short photoperiod reduced elongation of stretching internodes quantitatively, in a temperature-dependent manner.

**Fig. 2:**
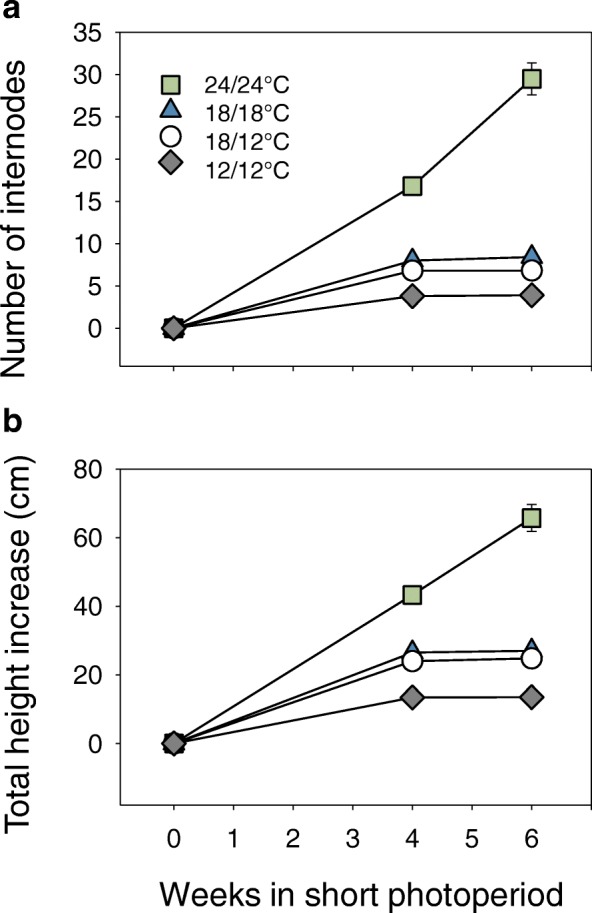
Elongation growth under short photoperiod and different temperatures. Plants were grown for six weeks under short photoperiod (10 h) under high (24/24 °C; D/N), moderate (18/18 °C; D/N), moderate/low (18/12 °C; D/N) and low temperatures (12/12 °C; D/N). **a** Number of new internodes produced per time point. **b** Overall increase in shoot height. (values are means ± SE, most smaller than symbols; *n* = 15 per data point)

The incipient and newly formed internodes showed a similar but much stronger trend. At constant standard temperature (18/18 °C), elongation of the top three internodes diminished by approx. 40% relative to the internodes below it (Fig. 1). When only the night-temperature was lower (18/12 °C) the reduction exceeded 70% (Fig. 1). Lowering also the day-temperature (12/12 °C) reduced elongation slightly further (78%, Fig. 1). In brief, short photoperiod alone affected mostly the incipient and new internodes. Low temperature during the night amplified this effect, whereas continuing low temperature through the day hardly reduced elongation further, reflecting reduced photosynthesis and night-time transport of photosynthates to incipient internodes. Low temperature also significantly increased the plastochron. During night-time it slightly reduced internode production, while continuous low temperature halved it (Fig. 2a). Similarly, overall gain in height was halved by continuous low temperature (Fig. 2b).

The 24/24 °C temperature regime induced unique effects. In contrast to the other temperature treatments, internode stretching was the same in the two different internode categories. Notably, the incipient internodes lacked the enhanced responsiveness to short photoperiod, measured in the other temperature regimes (Fig. 1). In addition, the internodes that were already elongating remained significantly shorter at this high temperature, relative to similar internodes at moderate temperature (Fig. 1). Under high temperature the production and elongation of new internodes was prioritized, as the plastochron dramatically decreased, tripling the number of elongating internodes and leaves at week 4 (Fig. 2a).

### Pre-dormancy low temperature converts older leaf primordia to scales

We next investigated if low temperature advances short photoperiod-induced TB initiation, as proposed in the literature. The first morphologically visible sign of bud formation is the metamorphosis of leaf primordia into scales. During leaf development, a primordium produces a lamina as well as stipules, thread-like appendages that decorate the leaf-base. Under short photoperiod, the apex reverses its developmental priorities, blocking lamina and leaf development while enlarging and hardening the stipules into bud scales. Because the last emerging long photoperiod leaves wrap the apex, visually concealing scale formation, we used a validated method to reconstruct the timing of scale initiation [28]. For each of the selected photoperiod-temperature regimes, we counted the number of internode-leaf units that emerged up to the point where the SAM had initiated scale production. This internode-leaf unit count is analogous to the method used to determine flowering time in *Arabidopsis*.

**Fig. 3:**
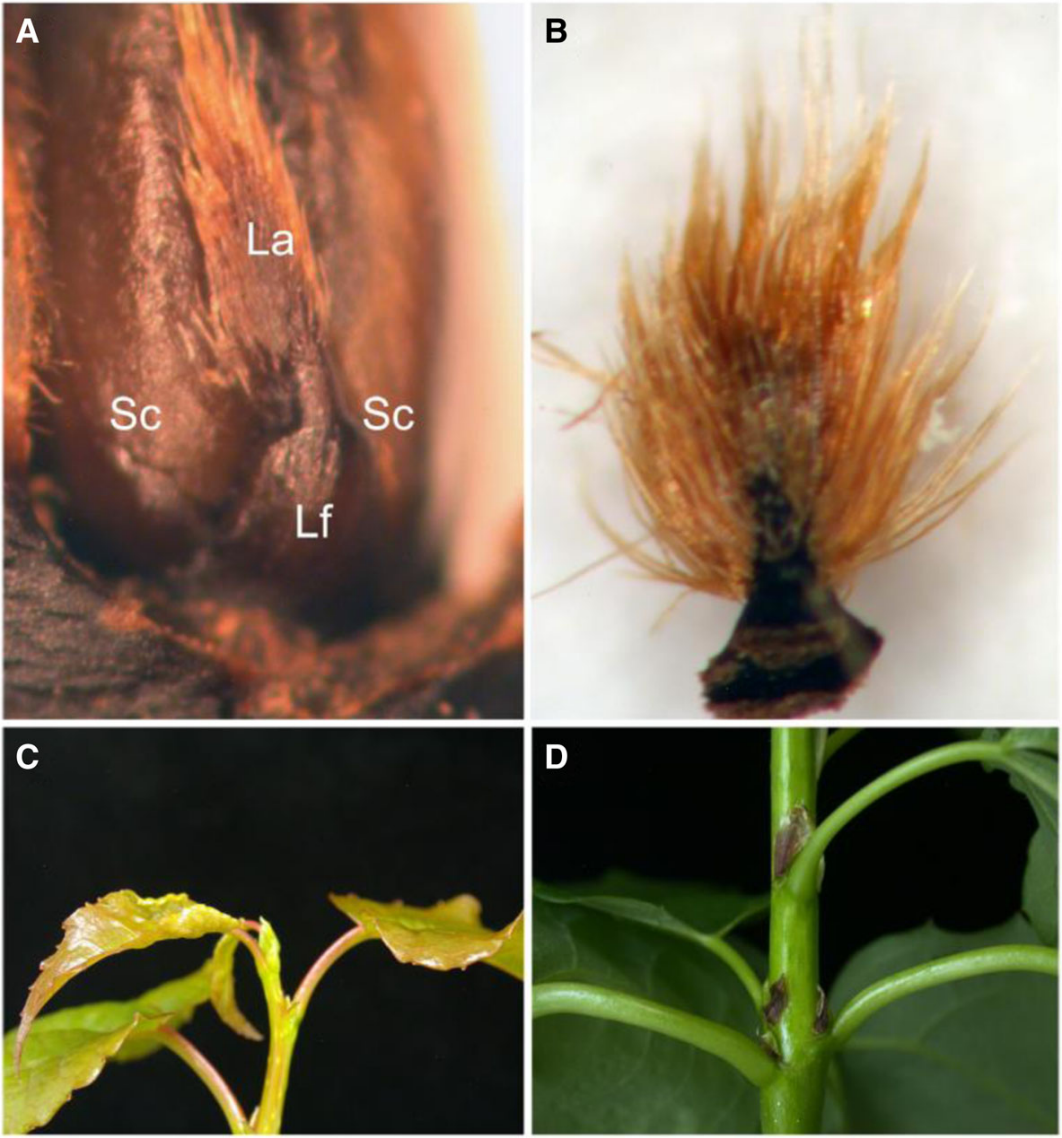
Terminal bud development at different temperature regimes. **a** Terminal bud scale with rudimentary lamina at continuous pre-dormancy low temperature (12/12 °C). **b** Detail of rudimentary lamina. **c** Terminal bud formation at continuous high temperature (24/24 °C); the scales are green. **d** Post-flushing elongated stem at continuous high temperature (24/24 °C). Brownish bud scales decorate the stem. Sc, scale; La, rudimentary lamina; Lf, leaf

This showed that relative to 18/18 °C, the plastochron was shortened at 24/24 °C and lengthened at 12/12 °C (Fig. 2a). At constant 12 °C, the overall production of primordia slowed down substantially. Consequently, the first scales arose from developmentally older primordia that had formed under long photoperiod (Additional file 1: Table S1). If left at 18/18 °C, these primordia would have developed into leaves. Indeed, these developmentally older scales often had a rudimentary lamina (Fig. 3a, b). Under moderate temperature (18/18 °C), such lamina between the scales was not observed. In brief, low temperature affected TB composition by producing bud scales from older leaf primordia (Additional file 1: Table S1).

### Pre-dormancy low temperature reduces embryonic shoot development

At low temperature (12/12 °C) the plastochron was lengthened (Fig. 2), resulting in a general slow-down of primordium initiation at the apex, and a reduction of the number of primordia that gave rise to embryonic leaves inside the emerging bud. TBs that developed under standard conditions (18/18 °C) possessed 10 embryonic leaves on average, while TBs that were exposed to the mildly low temperature typically possessed 7 embryonic leaves. Interestingly, it did not make a difference if low temperature was applied continuously (12/12 °C) or only during the night (18/12 °C), suggesting that also here night-time import via the phloem is a limiting factor. Young AXBs, below the apex, responded in a similar fashion, as the number of embryonic leaves was reduced from 10 to 8 at both temperature regimes.

**Fig. 4:**
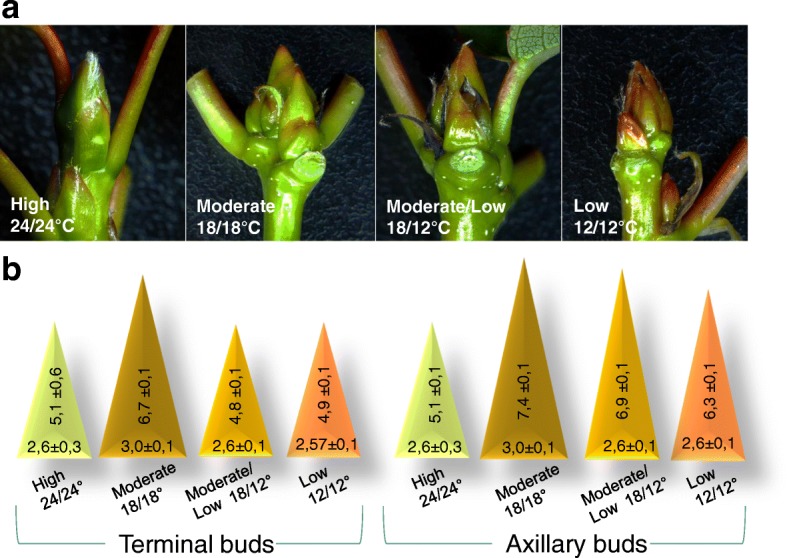
Bud development under short photoperiod at different temperatures. **a** Terminal buds at the indicated temperatures (at week 4). **b** Average lengths (vertical numbers) and widths (horizontal numbers) of terminal buds and axillary buds at the indicated temperature conditions (at week 6). Predormancy low temperature (12/12 °C) promoted anthocyanin production in both terminal and axillary buds, while buds at high temperature (24/24 °C) remained green. The relative color differences are indicated in the schemes. (values are means ± SE, *n* = 5 per data point)

As a result, TBs that formed at low temperature (either 18/12 °C or 12/12 °C), as well as AXBs that continued development at this temperature, were much smaller than those produced at moderate temperature (18/18 °C). TBs were clearly longer and broader at 18/18 °C than at the other temperature regimes. To a lesser degree, this was also true for AXBs (Fig. 4).

### Pre-dormancy low temperature reduces dormancy establishment

As low temperature adversely affected bud ontogeny and structure, we next investigated if it also affected their capacity to establish dormancy. We applied low temperature only at night (18/12 °C), or continuously (12/12 °C). Although less effective than 5 °C, we chose 12 °C because it is permissive of growth and development, while still being in the 5-12 °C range for chilling and vernalization.

**Fig. 5:**
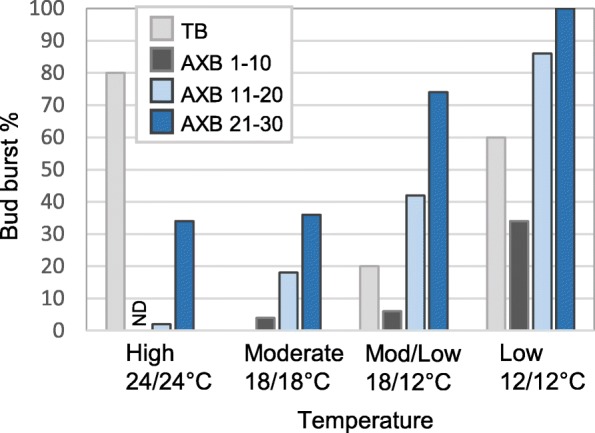
The effect of temperature on bud dormancy establishment. Dormancy in terminal buds (TB), in young developing axillary buds (AXB) at position 1–10 (counted from the top; black bar) and mature AXBs at positions 11–20 (light blue bar) and 21–30 (dark blue bar). Bud burst was monitored using single internode cuttings under long photoperiod and constant 18 °C. ND, no data collected due to default branching. Buds that did not burst under these conditions were considered dormant. (*n* = 5 plants; 50 AXB per data point)

At the moderate reference temperature (18/18 °C), it took four weeks to produce TBs, and after five weeks 100% of TBs and almost all AXBs (AXB positions 1–10) established dormancy (Fig. 5). Most AXBs at lower positions (11–30) had completed their development and were para-dormant prior to the transfer to short photoperiod. We found that low temperature during bud development significantly reduced dormancy establishment in both TB and AXBs (Fig. 5). The young AXBs were more resistant to the dormancy-releasing effect of low temperature than the TBs, reflecting that TBs were activated earlier than young AXBs. When only the night temperature was low, about 20% of TBs failed to establish dormancy, while this increased to approx. 60% when the temperature was continuously low (Fig. 5).

In young AXBs (positions 1–10), 12 °C at night-time alone hardly affected dormancy, but at continuous low temperature bud burst increased to 35% (Fig. 5). Older AXBs were highly responsive to 12 °C, and about 40% (11–20) and 75% (21–30) burst after night-time low temperature, and about 85% and 100% after continuous low temperature exposure, respectively (Fig. 5). The results clearly show that in combination with short photoperiod even mild chilling dampens dormancy establishment, demonstrating that in this species low temperature (12 °C) does not contribute to dormancy, but instead antagonizes it.

Interestingly, while low temperature dampens dormancy, continuous high temperature (24/24 °C) prevented dormancy establishment in TBs, as most of them flushed (80%) after 3 to 4 weeks under short photoperiod. Surprisingly, new TB were produced, but part of them flushed again (ca. 40%). Young AXBs, flushed simultaneously with the TBs, and produced branches.

### Pre-dormancy low temperature prematurely induces dormancy-release genes

To assess why short photoperiod failed to induce dormancy under low temperature in a large portion of TB (60%) and young AXBs (35%) (Fig. 5), we analysed the expression of genes that are known to function in chilling-induced dormancy release [40]. We hypothesized that the antagonizing effect of low temperature was due to a prematurely triggered, mistimed chilling response that engages part of the molecular machinery that releases dormancy. To test this, we investigated gene expression in young developing AXBs, which were exposed to short photoperiod at different temperatures. We analysed the expression of genes involved in the production of biologically active GAs and GA signalling, as well as GA deactivation. In addition, we analysed the expression of four 1,3-β-glucanase genes (GH17), encoding representative members of the GA_4_-responsive α- and GA_3_-responsive γ-clades.

**Fig. 6:**
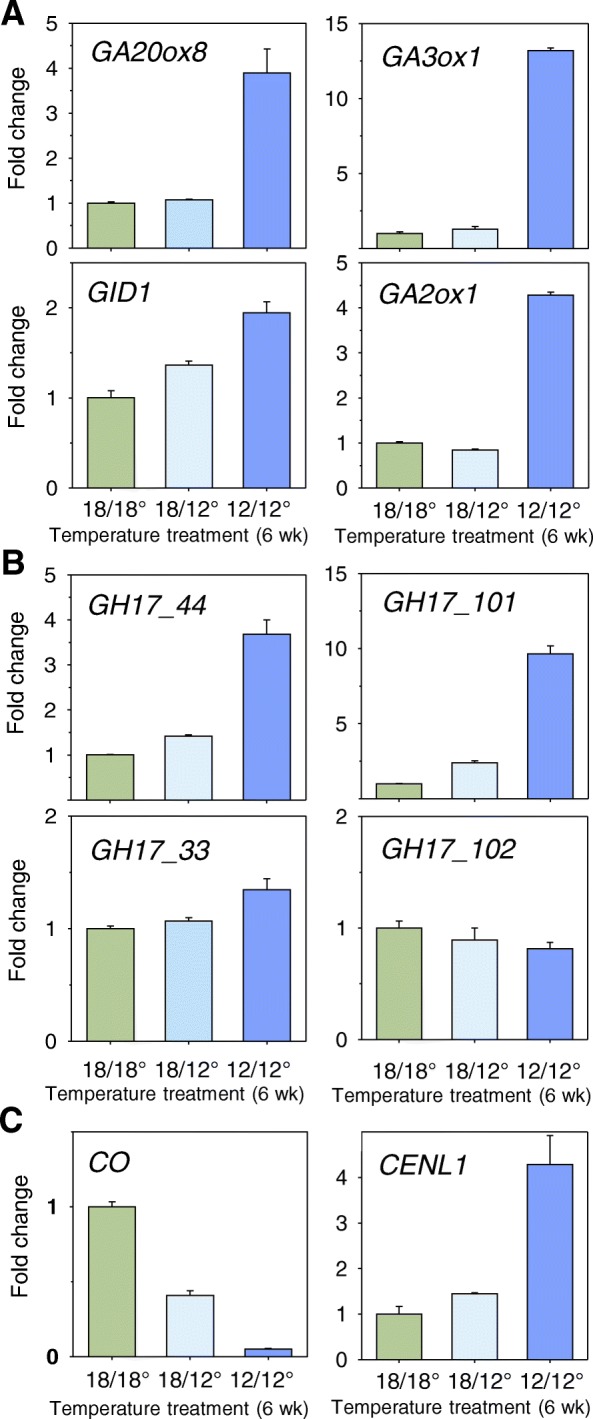
Effect of combined temperature-short photoperiod treatment on the expression of selected (**a**) gibberellin pathway genes *GA20ox8, GA3ox1*, *GID* and *GA2ox1,* (**b**) 1,3-β-glucanase genes *GH17_44, GH17_101, GH17_33* and *GH17_102*, and (**c**) flowering time genes *CO* and *CENL1*. Temperature regimes: moderate (18/18 °C), moderate/low (18/12 °C) and low (12/12 °C). Data represent gene expression in young axillary buds after six weeks. (values are means ± SE, *n* = 6 per data point)

Our data show that *GA20ox8* and *GA3ox1*, key members of the GA20ox and GA3ox families, which are involved in the two final steps in the production of bioactive GAs [56], were considerably upregulated in AXBs that developed under low temperature (Fig. 6a). Similarly, *GID1*, which encodes a GA-receptor, was upregulated by low temperature in a dose-dependent manner (Fig. 6a). *GA2ox1*, encoding a GA-deactivating enzyme, was also upregulated by low temperature (Fig. 6a).

We previously demonstrated that during chilling-induced dormancy release various members of the 1,3-β-glucanase enzyme-family localize to plasmodesmata where they hydrolyse callose at DSC, thereby potentiating cell-cell metabolic coupling, transport and communication [40]. The present data show that the GA_3_-inducible γ-clade 1,3-β-glucanase genes, *GH17_44* and *GH17_101*, were considerably upregulated under continuous low temperature (Fig. 6b). In contrast, the structurally different α-clade 1,3-β-glucanase genes, *GH17_33* and *GH17_102,* which possess a GPI-anchor for localization to the outer leaflet of the plasma membrane, remained relatively unaffected by the low temperature (Fig. 6b).

In brief, all the studied genes involved in GA-biosynthesis and GA-signalling, as well as those encoding 1,3-β-glucanases, showed an expression pattern during pre-dormancy low temperature treatment that resembled their expression during chilling-induced dormancy release at 5 °C [40] (Additional file 1: Figure S1).

### Pre-dormancy low temperature modifies expression of floral pathway genes

**Fig. 7:**
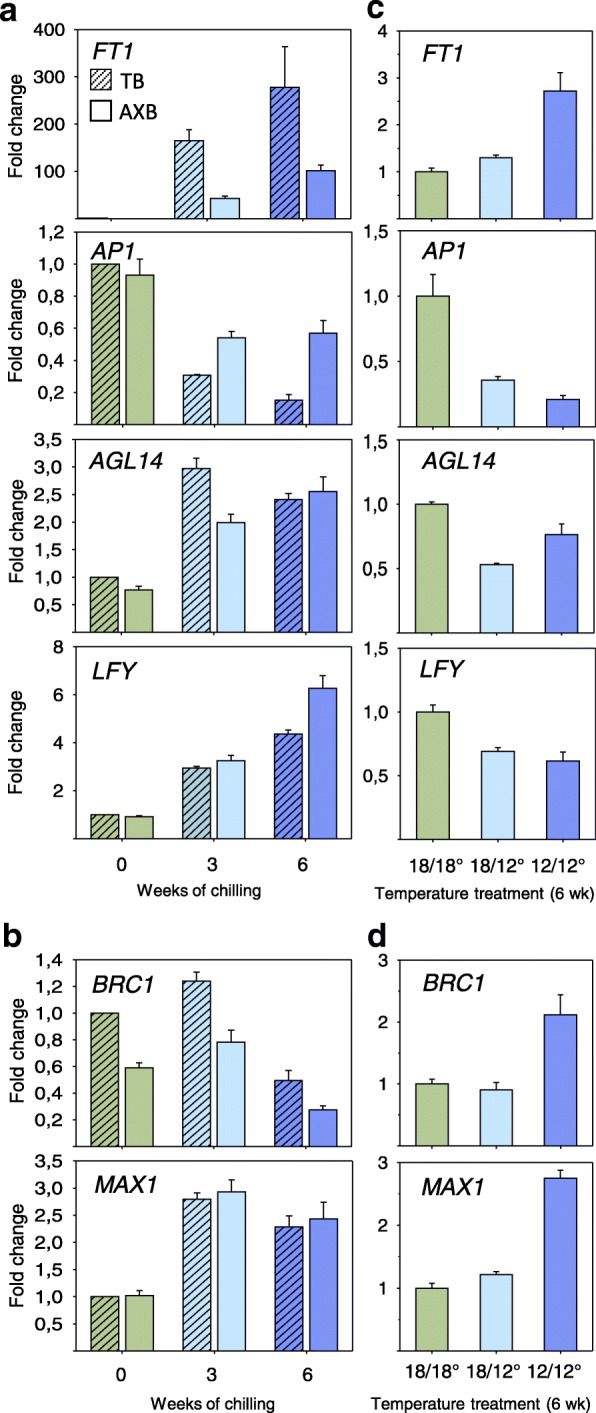
Effect of sequential (**a**, **b**) and combined (**c**, **d**) treatment with short photoperiod and temperature on the expression of selected (**a**, **c**) flowering time genes, *FT1*, *AP1*, *AGL14* and *LFY*, and (**b**, **d**) branching inhibitory genes, *BRC1* and *MAX1*. **a**, **b** Expression in terminal buds (TB), and in young axillary buds (AXB) after zero, three and six weeks chilling (5 °C). Plants at point zero (no chilling) were dormant after six weeks of short photoperiod. **c**, **d** Expression in young axillary buds after a 6-week period at the indicated temperatures (day/night): moderate (18/18 °C), moderate/low (18/12 °C) and low (12/12 °C). (values are means ± SE, *n* = 6 per data point)

The finding that pre-dormancy low temperature hampered bud ontogeny and dormancy establishment suggests that it might also affect gene expression related to vernalization, even though these young poplars are not yet competent to flower. Notably, some of these genes, including *CO*, *CENL1,* and *FT*, play a role in both dormancy cycling and floral transition. To assess this, we analysed the expression of these three key floral pathway genes. The present data show that pre-dormancy low temperature significantly reduced *CO* expression in a dose dependent manner (Fig. 6c). Continuous low temperature prevented the downregulation of *CENL1* (Fig. 6c), although during short-photoperiod induced dormancy establishment it is typically downregulated in TBs and AXBs. *FT1* expression was virtually absent from dormant TB and AXBs, but it was hyperinduced after six weeks of chilling (5 °C), whereas the pre-dormancy low temperature upregulated it relatively little (Fig. 7a, c).

We hypothesized that other floral pathway genes downstream of *CO*/*FT* could also be differently affected by pre-dormancy low temperature. Therefore, we investigated three key floral pathway genes that are related to vernalization, including putative poplar homologs of the *SOC1*-clade gene *AGAMOUS-LIKE 14* (*AGL14*), *APETALA1* (*AP1*), and *LEAFY* (*LFY*). Expression of *AGL14* was lowest in dormant buds, but increased within three weeks in both TBs and AXBs after chilling at 5 °C (Fig. 7a). In contrast, pre-dormancy low temperatures reduced *AGL14* expression in comparable AXBs to about half in the same period (Fig. 7c). *AP1* expression during chilling of dormant buds was reduced, particularly in the TBs (Fig. 7a). During pre-dormancy, low temperature also significantly reduced *AP1* expression in AXBs (Fig. 7c).

Considering that *AGL14* induces *LFY*, it was not unexpected that the pattern of *LFY* expression was similar to that of *AGL14* in chilled dormant buds (Fig. 7a). Levels of *LFY* transcripts were highest at week six, at which point dormancy is released, in both TBs and AXBs (Fig. 7a). In stark contrast, pre-dormancy low temperature significantly reduced *AGL14* and *LFY* expression, relative to the expression in chilled dormant buds (Fig. 7c). In conclusion, the upregulation of *FT1*, *AGL14* and *LFY* by low temperature occurs only in dormant buds.

**Fig. 8:**
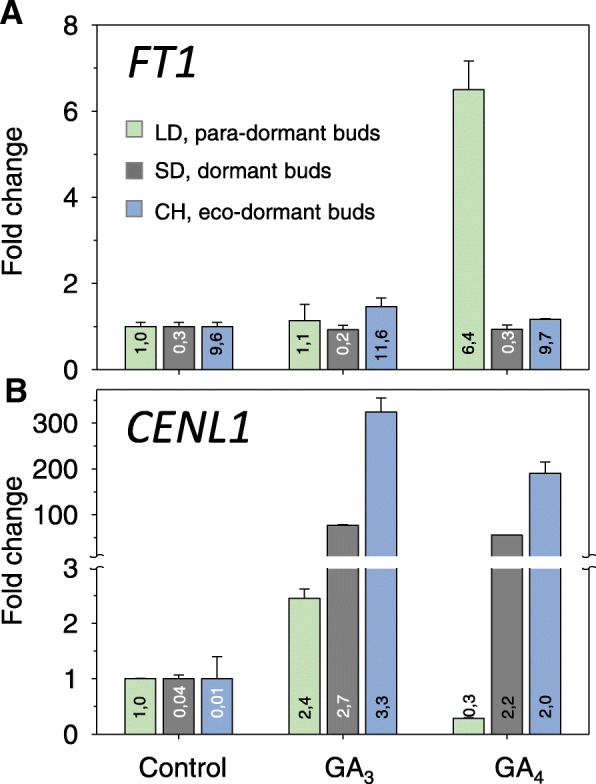
Effect of gibberellin GA_3_ and GA_4_ on the expression of (**a**) *FT1*, and (**b**) *CENL1*, at different stages of the dormancy cycle in hybrid aspen. Water with or without GAs (100 μM) was xylem-fed into the buds for three days, at which time-point bursting was not yet visibly initiated. The bars show fold changes relative to the water control of each dormancy stage (set at 1). The numbers on the bars show differences in expression levels between the different stages. (values are means ± SE, *n* = 6 per data point)

Contrary to buds, *FT1* transcripts in leaves were not at a measurable level regardless of the temperature regime. Surprisingly, the embryonic leaves that emerged from the buds after TB flushing at 24/24 °C expressed *FT1* at steady, albeit relatively low levels (Additional file 1: Figure S2). Considering that xylem-fed GA_4_, but not GA_3_, induces bud flush even when buds are dormant [40], we investigated if these hormones could potentially mediate high temperature-induced bud flushing. This showed that GA_4_, but not GA_3_, promoted *FT1* expression in para-dormant buds (Fig. 8a). In contrast, while GA_3_ promoted *CENL1* expression in all bud types and states, GA_4_ did not promote it in para-dormant buds (Fig. 8b).

### Pre-dormancy low temperature increases the expression of branch inhibitor genes

The bud burst test showed that not all buds were able to burst after pre-dormancy low temperature treatment. Gene expression analyses showed that the genes that are involved in bud suppression were upregulated, possibly contributing to diminished bud break. Suppression of AXB burst in hybrid aspen involves *MORE AXILLARY BRANCHES* (*MAX1*), a strigolactone biosynthesis gene, and *BRANCHED1 (BRC1)*, encoding a transcription factor that functions downstream of MAX1. As shown here, six weeks of chilling and pre-dormancy low temperature upregulated *MAX1* two to three-fold (Fig. 7b, d). In contrast, *BRC1* expression was reduced after six weeks in chilled dormant buds, whereas pre-dormancy low temperature upregulated the gene (Fig. 7b, d). In conclusion, the expression of these branch-inhibitor genes could counteract the dormancy-releasing effect of pre-dormancy low temperature, thereby preventing flushing.

### Timing of dormancy at distinct photo- and thermoperiods

To investigate if our finding that pre-dormancy low temperature antagonizes dormancy establishment (Figs. 5 and 6) is in agreement with the situation in the natural environment, we analysed data of European aspen, a close relative of hybrid aspen. Populations indigenous to latitudes 63^o^, 59^o^, 56^o^ and 51^o^ have a critical photoperiod of 21 h, 19 h, 17 h and 15 h, respectively [25]. We compared the length of the local photoperiod with the average monthly as well as daily minimum and maximum temperatures at the given coordinates. The publicly available data (https://www.timeanddate.com) show that the critical photoperiod (day length) for the two northernmost ecotypes is at the June solstice, whereas for the two southernmost ecotypes, the critical photoperiods are perceived in mid-July and early August, respectively (Additional file 1: Figure S3). As the development of complete TBs takes about four weeks [28], bud formation in all ecotypes appears to be completed before temperatures reach sustained low levels.

## Discussion

The perennial growth habit of deciduous trees owes its success to a strategy that maximizes the growing period, while leaving sufficient time to prepare for winter. This proactive program implements a sequence of events that culminates in the establishment of dormant acclimated buds. Historically, dormancy is an umbrella concept that covers multiple inactive states, attributed to whole plants, organs, or any meristem-containing structure [19, 57]. Two decades ago, a new paradigm was proposed, identifying dormancy as an intrinsically arrested state, the locus of which is the SAM [44, 45]. This anchors the concept of dormancy to the SAM as a self-organizing and autopoietic unit that drives primary morphogenesis, and self-arrests in response to short photoperiod signalling [44–48]. Escape from self-arrest requires chilling [16, 58]. The orderly progression of the dormancy cycle, guided by a sequentially declining photo- and thermoperiod, might be perturbed by the thermal instabilities that accompany global warming. The present experiments demonstrate that pre-dormancy low temperature provides chilling to the buds, because it triggers the dormancy release mechanism, validating the concept of pre-dormancy chilling.

### Climate change may affect dormancy establishment

Despite the fact that global temperatures have been rising, and heat waves have become more frequent and long lasting during the past decades [59, 60], the effect of temperature on dormancy cycling in perennials has not received the attention it deserves. Although warming is often viewed as positive, potentially lengthening the growing period and yield, it can also have negative effects due to the interaction with other environmental parameters. The present study confirms this scenario. Although high temperature significantly increased growth, in combination with short photoperiod it led to a reduced capacity to establish dormancy. Due to warming, trees will initiate TBs at increasingly higher temperatures at their current locations, potentially compromising dormancy. In a previous study on *Populus*, dormancy establishment at 25 °C was strongly delayed [61]. In the current experiments most buds failed to establish dormancy, and buds flushed, even repeatedly (Fig. 3d). Although high temperatures compromise the capacity to establish dormancy, flushing can be a sign of adaptive plasticity. For example, it may enable utilization of the full growing season in situations where very early bud burst leads to pre-mature dormancy before the June solstice [62].

A surprising finding was that *FT1* was involved in high temperature-induced (24 °C) bud flushing (Additional file 1: Figure S2). As GA_4_ induces bud burst [40], and is required for elongation growth, it is tempting to speculate that a network including FT1 and GA-regulated 1,3-β-glucanases drives high-temperature induced premature bud flushing. Meristem transitions triggered by temperature elevation could be more general, as in *Arabidopsis* a moderate increase from 23 °C to 27 °C upregulated *FT* and induced flowering under short photoperiod [63].

### Pre-dormancy chilling does not advance TB initiation

Pre-dormancy chilling did not advance TB formation in our experiments, contrary to many literature suggestions [22–27]. These studies relied predominantly on visual observations. Indeed, TBs can become visible earlier, but we conclude based on bud ontogeny and scale development [64, 65] that TB visibility is not an adequate criterion for the timing of bud initiation. The reason is that low temperature slows down the production of primordia at the apex, possibly exposing older primordia to scale-inducing signals, and belatedly converting them to scales equipped with a rudimentary lamina (Fig. 3). Consequently, fewer leaves below the bud need to unfold, thereby revealing the completed TB earlier. Indeed, an earlier phenological study reported that low temperature does not affect the timing of bud set in the Swedish aspen collection [66]. Our data are in agreement with this, but show in addition that low temperature in the chilling range negatively affects bud ontogeny and development.

That pre-dormancy chilling increased the plastochron, reducing the number of embryonic leaves and bud size (Fig. 4), is disadvantageous because the embryonic shoot is a ‘lifeline through winter’, bridging the gap between seasons [54]. Surprisingly, this quality aspect of bud development has not received much attention, although it affects tree performance and survival. A reduced number of embryonic leaves might influence how well the bud can support production of neoformed leaves in the next season. In adult trees, which have many determinate shoots, and which do not produce neoformed leaves [67, 68], the quality of buds is even more important. In addition, it could also negatively affect floral competence, as reproductive meristems are formed in the axils of late pre-formed leaves in the AXBs [39, 42].

### Pre-dormancy chilling impedes dormancy establishment

**Fig. 9:**
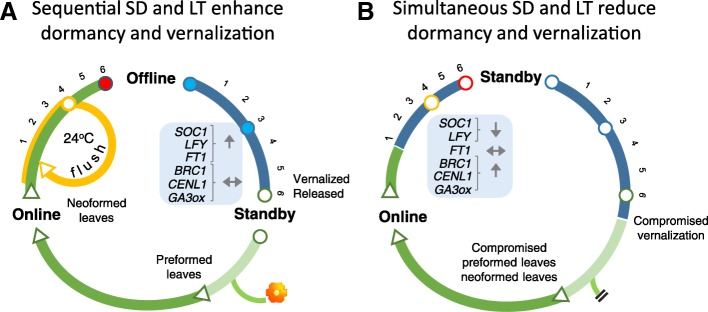
Temperature-dependency of the cell communication network during the dormancy cycle at the shoot apical meristem. **a** At moderate growth temperatures (dark green line, 18 °C), exposure to short photoperiod (SD) for 6 weeks (numbers on left) switches cells from an ‘online’ to an ‘offline’ state. Both terminal (TB) and axillary buds (AXB) have by then a complete embryonic shoot and a SAM that has established dormancy (red dot). At high ambient temperatures (orange line, 24 °C), buds may flush repetitively after reaching a hypothetical checkpoint (at 3–4 weeks; open orange dot). A 6- week exposure to low temperature (LT) (blue line, numbers on the right, 5-12 °C) releases dormancy and switches the meristem to a connected ‘standby’ state. A rise in temperature (light green line, 18 °C) induces burst, unfolding of preformed leaves, and production of neoformed leaves. AXBs of mature trees can produce flowers. Chilling characteristically regulates meristem-specific as well as floral and gibberellin pathway genes (light blue box). **b** Exposure to short photoperiod at low temperature (blue line, 12 °C) hampers bud ontogeny and all subsequent events in the bud dormancy cycle. It promotes a premature‘stand-by’ state and loss of vernalization by perturbing the expression of central genes (light blue box). Key: closed colored dots indicate dormant buds; open dots indicate nondormant buds; arrowheads indicate meristem proliferation; arrows in the light blue boxes indicate up- or downregulation of genes; horizontal double arrow indicates absence of clear effects

An important focus of the current work was to assess if low temperature affects dormancy establishment. During dormancy, callose containing DSC at all plasmodesmata lock the SAM in a dormant ‘offline’ state, in which the cell-cell communication that drives morphogenesis is abolished [46, 49] (Fig. 9). The molecular mechanism of chilling-induced dormancy release restores the symplasmic communication network involving plasmodesmata-associated 1,3-β-glucanases, which hydrolyse callose at the plasmodesmal DSCs [16], creating a ‘standby’ state that is inactive and eco-dormant. Developing AXBs and TBs can become dormant under short photoperiod, probably because as active sinks they are accessible to long-distance photoperiodic signals [28, 29]. Although lower temperatures than the one used here during pre-dormancy chilling (12 °C) are more effective in providing chilling [69, 70], our findings show that this temperature reduced dormancy in a dose-dependent manner (Fig. 5). Despite being at the border of the chilling range [35–37], this temperature had a cumulative effect like chilling-induced dormancy release at 5 °C [16]. Importantly, our molecular analyses indicated that it also utilized the same mechanism as in chilling-induced dormancy release [40]. Our results do not exclude that low temperature and other stresses, like drought, can induce growth cessation and even bud formation [12, 19, 30], but based on the present work these buds are likely to be eco-dormant.

Pre-dormancy chilling specifically and significantly upregulated the GA_3_-regulated γ-clade 1,3-β-glucanases, like in chilled dormant buds, while growth-related GA_4_-regulated α-clade 1,3-β-glucanases were hardly affected (Fig. 6b). This strongly suggests that under short photoperiod low temperature triggers this mechanism by default. As during chilling-induced dormancy release these GA_3_-regulated γ-clade 1,3-β-glucanases are delivered by lipid bodies to plasmodesmata [40], it seems likely that pre-dormancy chilling induces the same process prematurely.

The enhanced expression of the GA_3_-regulated 1,3-β-glucanases during pre-dormancy chilling is congruent with the simultaneous upregulation of the GA-biosynthesis and signalling genes (Fig. 6a). That GA-catabolism was also enhanced (Fig. 6a), like in dormancy release [40], could indicate that *GA2ox1* functions to deactivate growth-related GA_4_. The present findings suggest the possibility that pre-dormancy chilling triggers the premature involvement of the 13-hydroxylation pathway of GA biosynthesis, increasing the level of GA_3_.

That during pre-dormancy chilling buds failed to downregulate *CENL1* (Fig. 6a), which is characteristic of the dormant state [34], could be due to the elevated GA_3_ levels. Indeed, GA-feeding experiments showed that particularly GA_3_ strongly upregulated *CENL1* in dormant, para-dormant and released buds, while *FT1* was only upregulated by GA_4_ in para-dormant buds (Fig. 8). This contrasting induction of *CENL1* and *FT1* with different GAs suggests differential gene regulation and epigenetic states. The distinct nature of GA_3_- and GA_4_-induced effects on bud burst and gene expression is striking, and warrants further investigation.

Despite the activation of GA-biosynthesis, buds remain suppressed at low temperature, indicating that antagonistic factors are operational. A prominent inhibitor of bud activation is strigolactone [71]. Strigolactone biosynthesis gene *MAX1* and the strigolactone signalling gene *MAX2* are upregulated during AXB development, together with the downstream targets *BRC1* and *BRC2*, indicating that they collectively contribute to the suppression of buds in different *Populus* species [28, 72]. The current data show that pre-dormancy chilling further upregulates these inhibitory pathways (Fig. 7d). The strigolactone pathway is thus not only involved in suppressing burst of para-dormant AXBs, but also in repressing AXBs that are released from dormancy (Fig. 7b, d). That low temperature intersects with the strigolactone pathway might be more general, as it is also found in *Arabidopsis* and pea [73].

### Pre-dormancy chilling impedes vernalization pathway

There is an interesting parallel between vernalization and chilling-induced dormancy release. Both processes require similar low non-freezing temperatures over an extended period, and both processes are quantitative in nature [35–37]. Not surprisingly, it has remained a long-standing question if they involve overlapping processes [27, 36, 42, 43, 51, 74, 75]. During vernalization, floral suppressors diminish over time, allowing floral transition when the temperature rises in spring. For example, in *Arabidopsis*, a 6–12 week exposure to 4 °C epigenetically reduces the transcript level of the floral suppressor *FLOWERING LOCUS C* (*FLC*) in vascular tissue and the SAM, activating the genes *FT* and *SOC1* in leaves, and *SOC1* in the SAM [76–80]. Subsequently, the floral integrator *SOC1* and the mobile protein FT activate the floral identity genes *LFY* and *AP1* in the SAM, which can amplify each other’s expression [76, 81].

Although poplars do not have close homologues to *Arabidopsis FLC*, they do have *FLC-*like genes [82, 83]. It seems likely that also in poplar chilling removes an inhibitory module, as 5 °C-chilling of dormant buds hyperinduces *FT1.* This could involve *CO*, which encodes a B-box protein that binds to the *FT* promoter in a complex with AS1 [84]. *CO* expression in dormant buds of hybrid aspen increases during chilling, but in contrast, pre-dormancy chilling significantly reduced it (Fig. 6). This is congruent with the fact that pre-dormancy chilling did not upregulate *FT1* to the high level found during chilling of dormant buds (Fig. 7a, c). In adult poplar trees, *FT1* upregulation and acquisition of floral competence promotes floral transition [39, 41, 42].

In *Arabidopsis*, the floral transition involves *FT1*, *LFY* as well as *AGL14* [85]. We used putative *P. trichocarpa* homologues of these genes as indicators of the floral transition. Surprisingly, we found that hyperinduction of *FT1* during chilling of dormant buds was accompanied by a significant upregulation of the *AGL14* and *LFY* homologues in both TBs and AXBs. In contrast, pre-dormancy chilling reduced the expression of *AGL14* and in particular *LFY* (Fig. 7a, c). In *Arabidopsis*, *AP1* is required for petal and sepal development [86], while in aspen it is also upregulated under short photoperiod in developing poplar buds [33], possibly serving scale formation. Although chilling downregulated *AP1* expression in dormant buds as well as in chilled pre-dormant buds (Fig. 7a, c), in the latter case *AP1* transcription remained repressed throughout bud development. In conclusion, chilling of dormant buds promotes the expression of key floral pathway genes, whereas pre-dormancy chilling hampers their transcription (Fig. 7a, c). The implication might be that low temperature in the bud formation phase reduces floral competence. Thus, vernalization-induced flowering might require a previous dormancy phase, the realization of which might help to identify determinants of vernalization.

Despite the activation of floral pathway genes in chilled dormant buds, juvenile poplars do not transition to flowering, suggesting that inhibiting factors are present, including CENL1/TFL1. The balance of FT/TFL1 is believed to determine multiple aspects of growth and flowering [42]. For example, in young *Arabis alpina* plants, TFL1 competes with FT1 for binding to FD in a complex with 14-3-3 protein, thereby preventing vernalization [79]. Since in hybrid aspen, *CENL1/TFL1* expression ceases during dormancy establishment, and remains unaltered during dormancy release by chilling [40], additional floral inhibitors must be present. Particularly so, as *FT1* is upregulated in chilled dormant buds, and the FT1/CENL1 ratio should strongly favor flowering. These inhibitors could include BRC1, which in *Arabidopsis* interacts with FT to suppress FT-induced floral transition in AXBs [87]. Given the relative closeness of the two species [68], it seems possible that in juvenile hybrid aspen the high level of *BRC1* expression in AXBs developing under long photoperiod [28] and during pre-dormancy chilling under short photoperiod (Fig. 7d) can be involved in neutralizing *FT1*. Remarkably, pre-dormancy chilling not only failed to upregulate *FT1*, *AGL14* and *LFY*, it also enhanced expression of genes that repress bud burst or floral transition, including *MAX1, BRC1* and *CENL1*.

### Temperature-window for dormancy establishment

The present experiments, carried out in controlled conditions, demonstrate that bud formation and dormancy establishment benefit from moderate temperature conditions (around 18 °C). The decoupling of the photo- and thermoperiod during global warming might also have repercussions for trees in the field. For example, transplanted southern species are at risk of premature exposure to low temperatures, as they will initiate TBs at the critical photoperiod of their origin. In our controlled environment experiments, pre-dormancy chilling reduced bud quality, dormancy establishment and bud fate. High temperatures (24 °C) were equally disruptive, triggering bud flushing in the pre-dormancy phase. Although in nature, such day temperatures do occur during summer, they might be balanced by cooler nights [88]. Our experimental results suggest that sustained warm periods, as projected by climate models, will interfere with dormancy establishment.

The present finding that pre-dormancy chilling is an antagonizing force, is in agreement with the bud set data of closely related European aspen ecotypes (*P. tremula*) [25] (Additional file 1: Figure S3). The critical photoperiods of these provenances might have evolved under selection pressure to avoid low temperature exposure during bud development. However, it remains an interesting possibility that, with regard to temperature tolerance and sensitivity in dormancy cycling and vernalization, ecotypic variation might mitigate the effects of warming at high latitudes [89]. There can be standing genetic variation also in the length of the critical photoperiod, providing adaptive potential for northward migration without gene flow, except in the most northern populations [89].

In brief, locally adapted ecotypes secure proper bud development and dormancy establishment before temperatures decline to chilling levels. Thus, adverse temperature conditions, either too warm or too cold, may compromise dormancy establishment and reduce reproductive success. This is in agreement with early reports on *Populu*s species that photoperiodic induction of dormancy is effective only in a restricted temperature range [18, 19]. Although in general, fitness is a trade-off between investment in survival and reproduction, timely investment in winter preparation may serve both.

## Conclusions

Our experimental results, obtained in controlled environment conditions, support the hypothesis that photoperiodic induction of dormancy is successful within a 24-12 °C temperature-window (Fig. 9). This might also be a valid approximation for the natural environment. Even though early bud set seems to unnecessarily shorten the growing season, it serves the overall optimization of winter preparations and growth in the next season. At growth-promoting temperatures and relatively long photoperiods photosynthetic capacity and phloem transport can be maintained at a high level, allowing proper bud development, the filling of reserve stores, as well as the acquisition of freezing-tolerance and floral competence. Pre-dormancy chilling as well as unseasonal warm periods can impede the scheduled winter preparations, thereby reducing survival and reproductive success. We conclude that the mechanisms that induce and release dormancy operate simultaneously and antagonistically when plants are exposed to low temperature under short photoperiod. Global warming and erratic temperature patterns may therefore interfere with seasonal cycling and compromise overall fitness of photoperiodic ecotypes.

## Methods

### Plant material and experimental design

Hybrid aspen (*Populus tremula* x *P. tremuloides*) clone T89 was micropropagated in vitro, and planted in soil as described previously [41]. T89, which has a critical short photoperiod of 16 h [25], was grown at a long 18 h photoperiod in a greenhouse at approx. 18 °C and 75% relative humidity (RH). Plants were watered twice a day, and natural light was supplemented to ca. 200 μmol m^− 2^ s^− 1^ at 400 to 700 PPFD nm (Osram). After six weeks, when elongation and leaf production rates were constant, the approx. 80 cm tall plants were subdivided into two groups. One group (*n* = 72 plants) was exposed to short photoperiod for six weeks to induce dormancy. During this treatment TBs and developing AXBs (para-dormant) become dormant. Subsequently, plants were exposed for six weeks to traditional chilling at 5 °C to release dormancy (eco-dormant). The other group of plants (*n* = 80) were subdivided into four equal groups, transferred to controlled climate cabinets, and acclimated for three days in long photoperiod. Subsequently, four different day/night temperature regimes were used: ‘high’ (D/N 24/24 °C), ‘moderate’ (D/N 18/18 °C), ‘moderate-low’ (D/N 18/12 °C), and ‘low’ (D/N 12/12 °C). At the start of the distinct temperature regimes, the daylength was reduced to 10 h in all cabinets. Although temperatures between 5 and 7 °C are optimal for chilling induced dormancy release in many species, we chose 12 °C for low-temperature exposure because it is the upper temperature threshold for chilling while still permitting growth [35–37].

### Growth monitoring and bud structure analyses

We monitored plant growth by measuring overall shoot elongation as well as the number of new internodes that were produced at week four and six. After four weeks, we analysed bud structure in five plants per treatment. TBs and multiple AXBs were collected, fixed in 70% alcohol, and stored at 4 °C. Buds were examined, photographed and their sizes measured, using a preparation microscope. Each bud was opened with a surgical scalpel and the number of bud scales, embryonic leaves, and leaf primordia were counted. The presence or absence of a rudimentary lamina at the bud scales was recorded.

### Dormancy testing

We tested the effect of the different treatment regimens on dormancy establishment after six weeks by transferring TBs and bud-internode units to mild forcing conditions (16 h light, PPFD 170 μmol m^− 2^ s^− 1^, 18 °C, 70% RH). Stems were cut into internodes, with the AXB at the higher end of approx. 2 cm long segments. The stem bases were inserted through tiny holes in a supporting Styrofoam sheet, and floated on water or 100 μM solutions of GA_4_ or GA_3_, with the lower stem end submerged. Bud burst was scored as described previously [40]. AXBs were grouped into three subsequent categories, reflecting developmental-, mature- and potentially senescent-stages: ‘young AXB’ (positions 1–10 from the top), ‘maturing AXB’ (positions 11–20 from the top) and ‘old AXB’ (positions 21–30 from the top).

### RNA extraction and real-time qPCR analyses

To compare the effect of post-dormancy chilling and pre-dormancy chilling on gene expression two experiments were performed. (1) Dormancy was induced by short photoperiod at 18 °C, and subsequently plants were exposed to chilling. (2) Dormancy was induced by short photoperiod at four different temperature regimes (conditions as above).

We analysed TBs and young AXBs (positions 1–10), which are still sinks that are susceptible to short photoperiod signalling from the leaves, and can establish dormancy [28, 29]. For each data point, RNA was extracted from six plants, divided into two biological replicates. In all cases, sampling was carried out during the last hour of the short photoperiod. In addition, leaf tissue around major veins was collected from each group. Samples were frozen in liquid nitrogen and stored at − 80 °C for later RNA extraction and qPCR analysis.

Frozen leaf tissues and buds were ground with a mortar in 750 μl extraction buffer and processed further as described previously [29]. Quantitative reverse transcription-PCR (qRT-PCR) analyses were performed with the ABI Prism 7500 Fast sequence detection system, using SYBR Green PCR master mix (Applied Biosystems). Transcript levels were normalized with *Populus* actin. Gene-specific primer sequences are listed in Additional file 1: Table S2.

### Meteorological data and bioinformatics

Local photoperiod with the average temperature at the corresponding coordinates were retrieved from the publicly available website Time and Date (https://www.timeanddate.com).

BLAST searches in GenBank and the *P. trichocarpa* genome v3.0 [90] databases (http://www.ncbi.nlm.nih.gov/BLAST; http://www.phytozome.net) was used to identify genes, and ClustalW (http:// www.ebi.ac.uk/Tools/msa/clustalw2) to perform multiple sequence alignments. Gene- specific primer sequences for the qPCR analysis were designed with Primer3 (http://frodo.wi.mit.edu/primer3).

## Additional file

Additional file 1: **Table S1.** Bud scale-initiation date calculated from the internode-leaf units that emerged before the TB was formed. Days were deduced from the plastochron. **Figure S1.** Expression of gibberellin receptor, *GID1A* and *GID1B* during dormancy establishment under short photoperiod and subsequent dormancy removal by chilling. Expression is presented as fold changes using long day value as reference (=1). Plants were first kept for 8 weeks under short photoperiod (SD0-SD8) and subsequently chilled for 8 weeks (CH0-CH8) to remove dormancy. The end of short photoperiod treatment (SD8) is comparable to the beginning of chilling treatment (CH0). The approximate timing of dormancy establishment and dormancy removal in the given conditions are indicated by arrows. (values are means ± SE, *n* = 6). **Figure S2.** Expression of *FT1* and *FT2* in leaves under short photoperiod and at different temperature regimes. Plants were exposed to either high (24/24 °C), moderate (18/18 °C), moderate/low (18/12 °C) and low (12/12 °C) temperature conditions for six weeks. Expression was analysed in source leaves at all temperature combinations, and in the new leaves that unfolded after flushing at high ambient temperature (24/24 °C). Highest Ct values for *FT1* and *FT2* indicated. (values are means ± SE, *n* = 6). **Figure S3.** Photo- and thermo-periods of four European aspen ecotypes at their native locations. (A-D) Scatter plots (black line) indicate the length of the daily photoperiod, from 1 May to 31 September. The mean monthly temperatures are presented in the table below the plot, including absolute maximum and minimum of the given month, exemplified for the year 2010. The longest day, 21 June, is indicated by a green dot. The timing of bud burst varies at different latitudes, but at summer solstice the trees are actively growing at all locations. The first red dot indicates the date at which the days are clearly shorter (<) than the critical photoperiod of a given ecotype (data from [25]). Subsequent red dots indicate photoperiods that are shorter than the indicated number of hours. Once the short photoperiod is perceived, the formation of complete buds takes approx. 4 weeks [29]. Approximate timing of bud formation is indicated by red arrow. A blue dot indicates the approximate onset of low temperatures, although sporadically temperatures can drop also during the warm season. (E) Coordinates of the four latitudes (provenances). **Table S2.** Genes, model identifiers, and primer pairs for qRT-PCR. (PDF 3270 kb)
