# Supplementary material for: Decoupling photo- and thermoperiod by projected climate change perturbs bud development, dormancy establishment and vernalization in the model tree Populus
Source: BMC Plant Biol. 2018 Oct 5;18:220. doi: 10.1186/s12870-018-1432-0 (PMC6173867; doi:10.1186/s12870-018-1432-0)
Supplement: Supplementary file 1 — Table S1. Bud scale-initiation date calculated from the internode-leaf units that emerged before the TB was formed. Days were deduced from the plastochron. Figure S1. Expression of gibberellin receptor, GID1A and GID1B during dormancy establishment under short photoperiod and subsequent dormancy removal by chilling. Expression is presented as fold changes using long day value as reference (=1). Plants were first kept for 8 weeks under short photoperiod (SD0-SD8) and subsequently chilled for 8 weeks (CH0-CH8) to remove dormancy. The end of short photoperiod treatment (SD8) is comparable to the beginning of chilling treatment (CH0). The approximate timing of dormancy establishment and dormancy removal in the given conditions are indicated by arrows. (values are means ± SE, n = 6). Figure S2. Expression of FT1 and FT2 in leaves under short photoperiod and at different temperature regimes. Plants were exposed to either high (24/24 °C), moderate (18/18 °C), moderate/low (18/12 °C) and low (12/12 °C) temperature conditions for six weeks. Expression was analysed in source leaves at all temperature combinations, and in the new leaves that unfolded after flushing at high ambient temperature (24/24 °C). Highest Ct values for FT1 and FT2 indicated. (values are means ± SE, n = 6). Figure S3. Photo- and thermo-periods of four European aspen ecotypes at their native locations. (A-D) Scatter plots (black line) indicate the length of the daily photoperiod, from 1 May to 31 September. The mean monthly temperatures are presented in the table below the plot, including absolute maximum and minimum of the given month, exemplified for the year 2010. The longest day, 21 June, is indicated by a green dot. The timing of bud burst varies at different latitudes, but at summer solstice the trees are actively growing at all locations. The first red dot indicates the date at which the days are clearly shorter (<) than the critical photoperiod of a given ecotype (data from [25]). Subsequent red d [file 12870_2018_1432_MOESM1_ESM.pdf]

Additional file 1: Table S1

Additional file 1: Table S1. Bud scale-initiation date calculated from the internode-leaf units that emerged before the TB was formed. Days were deduced from the plastochron.

Number of plants	TB scale initiation date	Temperature (day/night)
22	0/21,5*	24/24ºC
19	0,4	18/18ºC
23	-1,2	18/12ºC
21	-4,1	12/12ºC

*Two dates due to flushing

Additional file 1: Figure S1

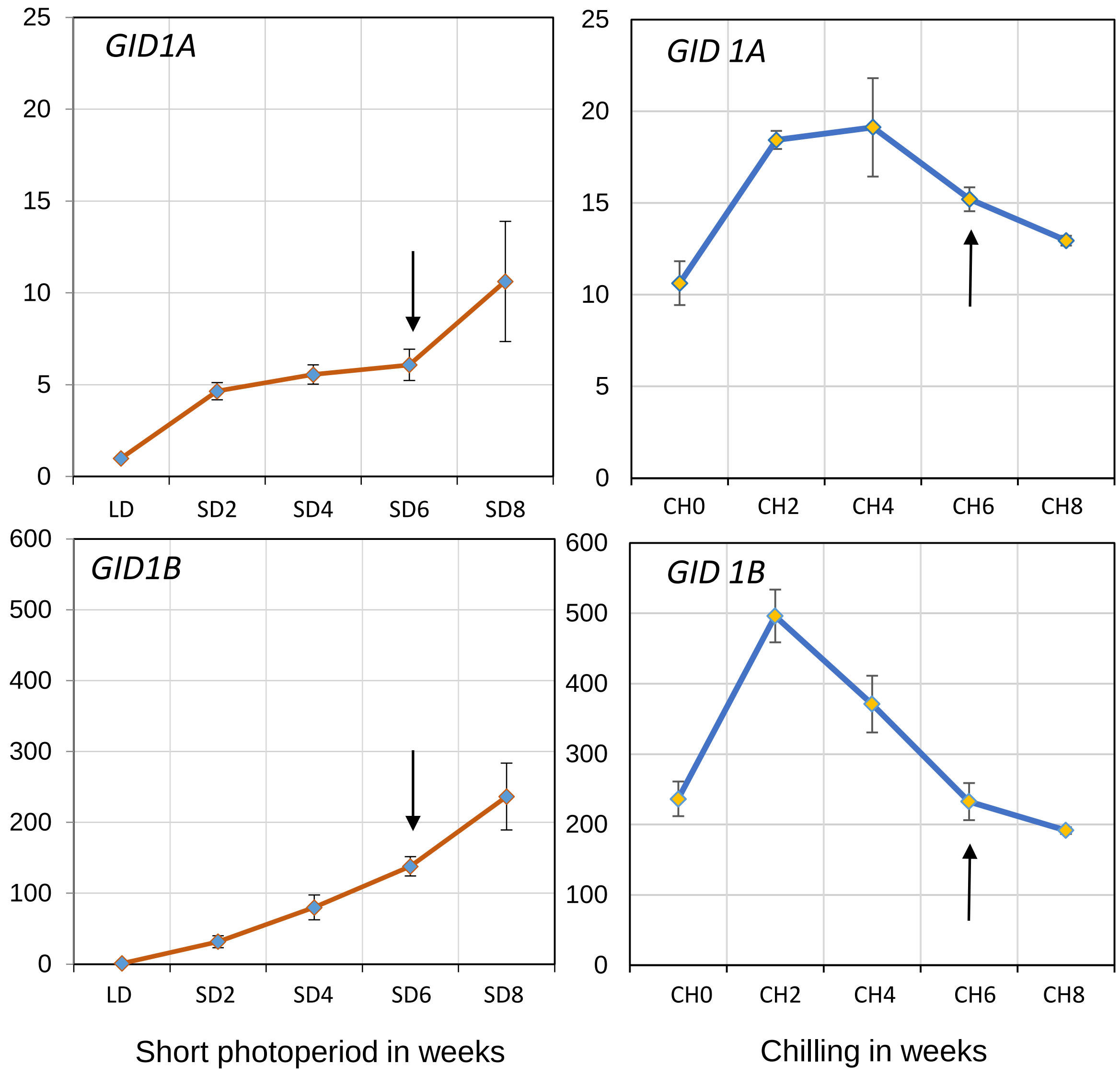

Additional file 1: Figure S1. Expression of gibberellin receptor, *GID1A* and *GID1B* during dormancy establishment under short photoperiod and subsequent dormancy removal by chilling. Expression is presented as fold changes using long day value as reference (=1). Plants were first kept for 8 weeks under short photoperiod (SD0-SD8) and subsequently chilled for 8 weeks (CH0-CH8) to remove dormancy. The end of short photoperiod treatment (SD8) is comparable to the beginning of chilling treatment (CH0). The approximate timing of dormancy establishment and dormancy removal in the given conditions are indicated by arrows. (values are means ± SE, n=6)

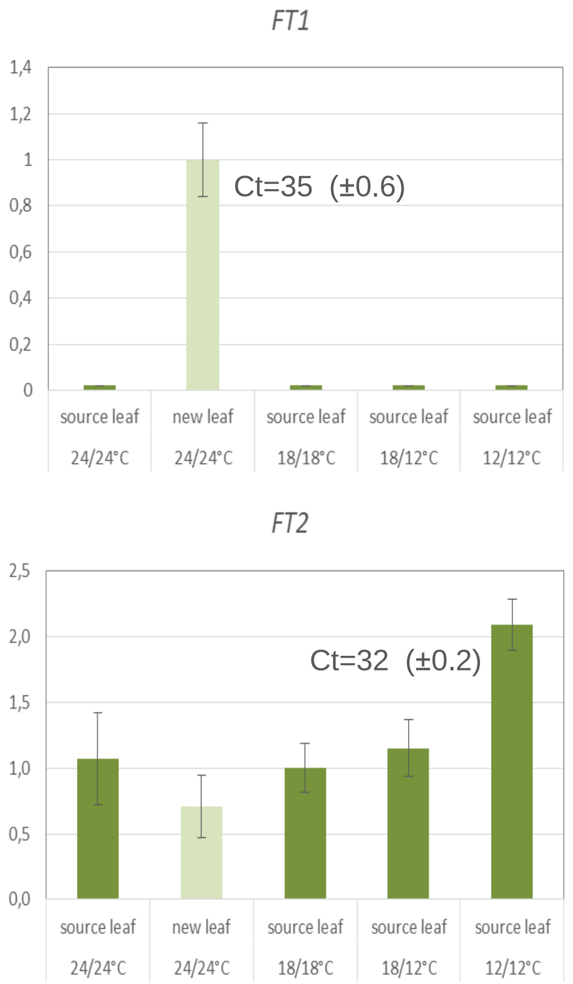

Additional file 1: Figure S2. Expression of *FT1* and *FT2* in leaves under short photoperiod and at different temperature regimes. Plants were exposed to either high (24/24°C), moderate (18/18°C), moderate/low (18/12°C) and low (12/12°C) temperature conditions for six weeks. Expression was analysed in source leaves at all temperature combinations, and in the new leaves that unfolded after flushing at high ambient temperature (24/24°C). Highest Ct values for *FT1* and *FT2* indicated. (values are means ± SE, n=6)

Additional file 1: Figure S3

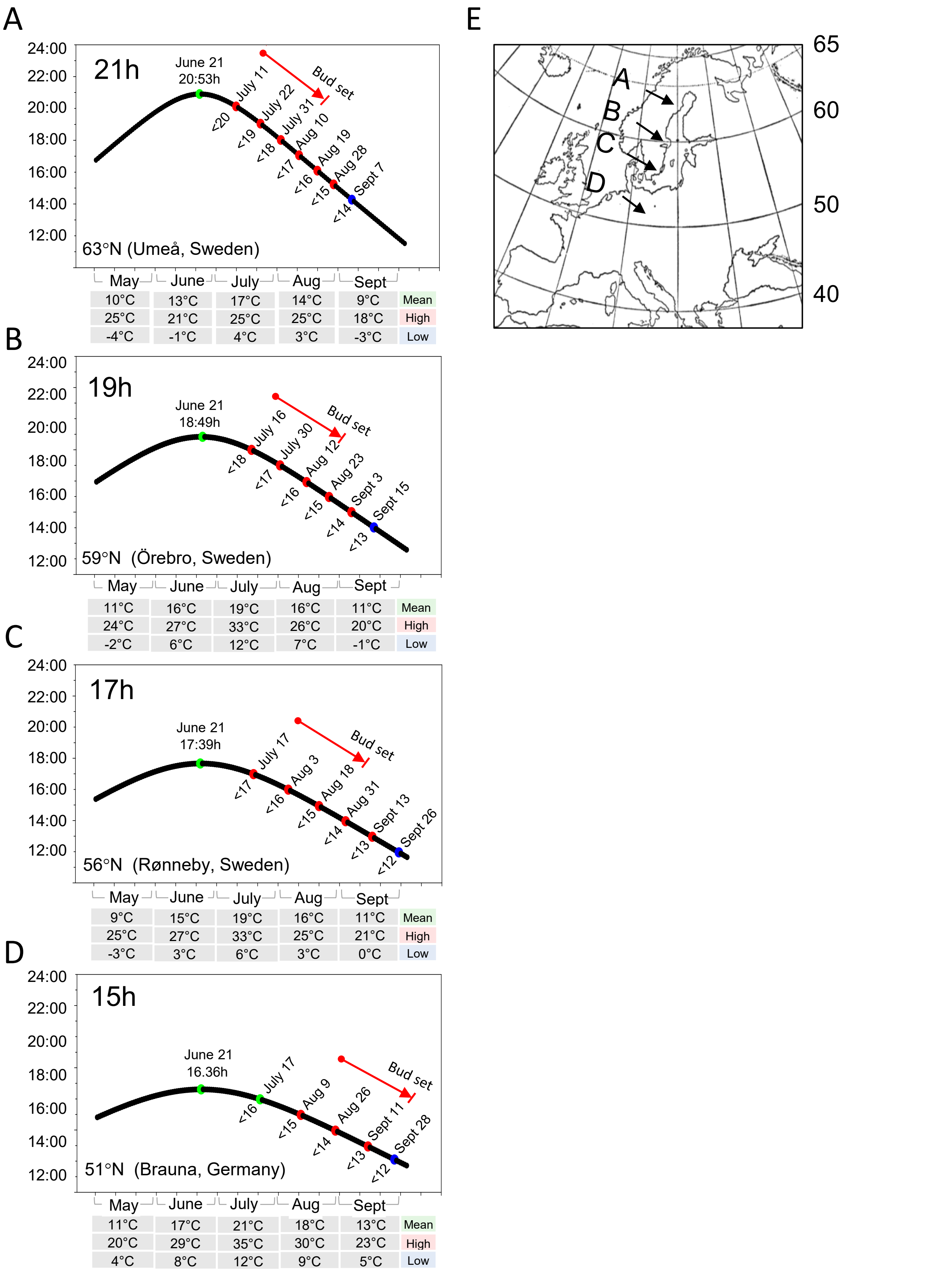

Additional file 1. Figure S3. Photo- and thermoperiods of four European aspen ecotypes at their native locations. (A-D) Scatter plots (black line) indicate the length of the daily photoperiod, from 1 May to 31 September. The mean monthly temperatures are presented in the table below the plot, including absolute maximum and minimum of the given month, exemplified for the year 2010. The longest day, 21 June, is indicated by a green dot. The timing of bud burst varies dependent on latitude, but at summer solstice the trees are actively growing at all locations. The first red dot indicates the date at which the days are clearly shorter (<) than the critical photoperiod of a given ecotype (data from [25]). Subsequent red dots indicate photoperiods that are shorter than the indicated number of hours. Once the short photoperiod is perceived, the formation of complete buds takes approx. 4 weeks [28]. Approximate timing of bud formation is indicated by a red arrow. A blue dot indicates the approximate onset of low temperatures, although sporadically temperatures can drop also during the warm season. (E) Coordinates of the four latitudes (provenances).

Additional file 1: Table S2. Genes, model identifiers, and primer pairs for qRT-PCR.

Protein	Gene	Populus trichocarpa v3.0	Primers	
		Locus name	Forward 5'-3'	Reverse 5'-3'
beta-1,3-glucanase	*GH17_33*	Potri.001G449100	CAATGCGTATCCCTTTTTCG	GGAAGTTGCTTTTCGGATCA
beta-1,3-glucanase	*GH17_44*	Potri.010G142800	TGCATTCTCCCCGAATAAAC	GGAAACTGTCGCGTTTTGTT
beta-1,3-glucanase	*GH17_101*	Potri.016G057600	AGAGAGGAACCCCAAAGAAG	AGAAAGATCCCCCAATGTTT
beta-1,3-glucanase	*GH17_102*	Potri.001G006500	TGCCATGAACTACCTCCACA	GGAAAGGCCTTGGGATAATG
GA3 oxidase 1	*GA3ox1*	Potri.001G176600	TGGCTCTCCTCTTGAGCATT	AACCATGTCAACCTCCTTGC
GA 20 oxidase 8	*GA20ox8*	Potri.015G134600	ATCAAAACCATGCCATCCA	TGGTGTCGAAGAACTTGTGC
GA2 oxidase 1	*GA2ox1*	Potri.001G378400	TTCTTCTCATTACCGCTCTCTG	TCTACCCAGCCCACATCAC
GIBBERELLIN INSENSITIVE DWARF1	*GID1A*	Potri.005G040600	ACCGTGGGACTAGCCTTCTT	ACAACCTCCGAGTTGACAGG
FLOWERING LOCUS T	*FT1*	Potri.008G077700	GCGAGCTCAAGCCCTCTCA	TGCATCAGGGTCCACCATAAC
FLOWERING LOCUS T	*FT2*	Potri.010G179700	GAGGTTGTGTGCTACGAGAGC	CACTGTTTGCCTGCCTAGTTG
CENTRORADIALIS-LIKE1	*CENL1*	Potri.004G203900	AGTCCAACAGGAAGCAGGTTTTT	AAAGGATCTCATATCACCTCCATGAA
CONSTANS	*CO*	Potri.004G108300	GATGTTGGAGTGGTGCCAGAA	TGGATAGCAGTGCTGGAGAAAAG
APETALA1	*AP1*	Potri.008G098500	TCAGTTACCAGGAAGAAGATCCA	TTCATGTTCCAAAGCATCCA
SUPPRESSOR OF OVEREXPRESSION OF CONSTANS1/AGL-14-related	*SOC1/AG14-related*	Potri.003G119700	AGTGTGGGATGCAACAACCT	TCTTATGGTTTTGGGGCAAG
LEAFY	*LFY*	Potri.015G106900	ATGCCCCACTAAGGTGACAA	AAGTGCGTTAGATGCGTCCT
MORE AXILLARY BRANCHES1	*MAX1.1*	Potri.006G226700	AAACGTTATGGCCCCATTTT	TGAGATGGGAGAGGGAACAC
BRANCHED1	*BRC1-LIKE*	Potri.012G059900	CATCATCGCGTAAAACTCCA	GTCGATTCTTCGACTGCACA
ACTIN	*ACT*	Potri.001G309500	CGATGCCGAGGATATTCAAC	ACCAGTGTGTCTTGGTCTACCC
